# Analytical and diagnostic characteristics of the high-sensitivity PATHFAST troponin I assay

**DOI:** 10.1186/cc14239

**Published:** 2015-03-16

**Authors:** E Spanuth, R Thomae, E Giannitsis

**Affiliations:** 1 DIAneering GmbH, Heidelberg, Germany; 2 Mitsubihi Chemical Europe, Düsseldorf, Germany; 3 University of Heidelberg, Germany

## Introduction

The POC PATHFAST cTnI assay (Mitsubishi Medience, Japan) has shown promising analytical validity. We thought to evaluate whether the assay could be classified 'highly-sensitive'.

## Methods

cTnI was determined using PATHFAST in 120 healthy individuals (60 men and 59 women, 21 to 69 years old, median 42 years). Cardiac disorders were excluded by cardiac magnetic resonance imaging. The diagnostic characteristics were investigated by determination of cTnI and cTnT (Roche hs-cTnT) in 181 patients admitted to the chest pain unit at presentation, and 3 and 6 hours later. The results were related to the discharge diagnoses.

## Results

The cTnI concentrations measured in the control group ranged from 0.4 to 17.2, mean 2.1 (95% CI: 1.6 to 2.6) ng/l, without age dependency. Men revealed higher levels than women, means (IQR) were 2.8 (1.2 to 2.6) and 1.1 (0.7 to 1.3) ng/l. The CLSI nonparametric method revealed a 99th percentile value of 16 ng/l. The quantification of cTnI above the LoD (1.0 ng/l) and below the 99th percentile was possible in 79 of 120 individuals. The imprecision profile according to NCCLS revealed 20%, 10% and 5% CVs at concentrations of 2, 3 and at 20 ng/l, respectively. The discharge diagnosis was NSTEMI in 71 patients. The cTnI median values at 0, 3 and 6 hours were 46, 166 and 399 ng/l, respectively. AUC values at 0, 3 and 6 hours were 0.923, 0.964 and 0.969 for hs-cTnT and 0.919, 0.962 and 0.958 for cTnI, respectively. cTnI revealed AUC values of absolute changes from admission to 3 hours and from admission to 6 hours were 0.920 and 0.931, respectively.

## Conclusion

The PATHFAST cTnI demonstrated complete fulfillment of the analytical criteria for high-sensitive cTn assays: The imprecision (CV) at the manufacturer-recommended 99th percentile value was 5%. The quantification of cTnI in was possible in 65.8% of healthy individuals. The examination of the diagnostic characteristics revealed complete concordance with the hscTnT assay for detection of NSTEMI and for assessment of absolute changes of cTn values (rise and/or fall) in NSTEMI patients. PATHFAST cTnI showed highly sensitive detection of NSTEMI with increasing sensitivity at admission and after 3 to 6 hours, not going along with decreased specificity. The PATHFAST cTnI might be useful at the point-of-care setting for early rule-in and rule-out diagnosis of NSTEMI.

